# The Deacetylase Sir2 from the Yeast *Clavispora lusitaniae* Lacks the Evolutionarily Conserved Capacity to Generate Subtelomeric Heterochromatin

**DOI:** 10.1371/journal.pgen.1003935

**Published:** 2013-10-31

**Authors:** Cara A. Froyd, Shivali Kapoor, Fred Dietrich, Laura N. Rusche

**Affiliations:** 1 Biochemistry Department, Duke University, Durham, North Carolina, United States of America; 2 Department of Biological Sciences, State University of New York at Buffalo, Buffalo, New York, United States of America; 3 Department of Molecular Genetics & Microbiology, Duke University, Durham, North Carolina, United States of America; University of California San Francisco, United States of America

## Abstract

Deacetylases of the Sir2 or sirtuin family are thought to regulate life cycle progression and life span in response to nutrient availability. This family has undergone successive rounds of duplication and diversification, enabling the enzymes to perform a wide variety of biological functions. Two evolutionarily conserved functions of yeast Sir2 proteins are the generation of repressive chromatin in subtelomeric domains and the suppression of unbalanced recombination within the tandem rDNA array. Here, we describe the function of the Sir2 ortholog ClHst1 in the yeast *Clavispora lusitaniae*, an occasional opportunistic pathogen. ClHst1 was localized to the non-transcribed spacer regions of the rDNA repeats and deacetylated histones at these loci, indicating that, like other Sir2 proteins, ClHst1 modulates chromatin structure at the rDNA repeats. However, we found no evidence that ClHst1 associates with subtelomeric regions or impacts gene expression directly. This surprising observation highlights the plasticity of sirtuin function. Related yeast species, including *Candida albicans*, possess an additional Sir2 family member. Thus, it is likely that the ancestral *Candida SIR2/HST1* gene was duplicated and subfunctionalized, such that *HST1* retained the capacity to regulate rDNA whereas *SIR2* had other functions, perhaps including the generation of subtelomeric chromatin. After subsequent species diversification, the *SIR2* paralog was apparently lost in the *C. lusitaniae* lineage. Thus, *C. lusitaniae* presents an opportunity to discover how subtelomeric chromatin can be reconfigured.

## Author Summary

The Sir2 deacetylases or sirtuins are an important family of proteins thought to regulate life cycle progression and life span according to nutrient availability. These proteins have diversified over the course of evolution such that humans possess seven family members and the laboratory yeast *S. cerevisiae* possesses five. By comparing the functions of sirtuins in various yeast species, we are gaining insight into how this family has diversified. Here, we studied the sirtuin called ClHst1 from *C. lusitaniae*, a yeast species known to cause infections in immunocompromised people. We demonstrate that although ClHst1 shares one function with its relatives in other yeast species, it has lost the capacity to act at telomeres, which are the ends of chromosomes. Thus, we have identified an opportunity to discover how organisms adapt to the loss of an evolutionarily conserved function.

## Introduction

The Sir2, or sirtuin, family of proteins is ubiquitous throughout all kingdoms of life [1], [2]. Members of this family catalyze the deacetylation of lysines, coupled to the consumption of the cofactor NAD^+^ to release nicotinamide, 2′-O-acetyl-ADP-ribose and the unacetylated lysine residue [3], [4]. The dependence of sirtuin activity on NAD^+^ has suggested a link between deacetylation and the metabolic state of a cell [5], [6]. Moreover, the biological consequences of sirtuin-mediated deacetylation imply a role for sirtuins in connecting life cycle progression and life span with nutrient availability [7], [8].

The genomes of most prokaryotic species encode a single member of the sirtuin family, whereas eukaryotic genomes encode multiple paralogs. For example, humans have seven sirtuins (SirT1-SirT7), and the yeast *Saccharomyces cerevisiae* encodes five (Sir2, Hst1-Hst4). This expansion can reasonably be explained by gene duplication followed by functional divergence of the duplicated genes. Based on phylogenetic analysis, sirtuins can be grouped into several subfamilies [1], [2]. The most-studied subfamily contains the founding member, yeast Sir2, as well as its ortholog, the mammalian SirT1. These proteins are nuclear and regulate chromatin structure and gene expression through the deacetylation of histones and transcription factors. A different subfamily contains SirT6 and SirT7, which are also nuclear and have chromatin-associated functions that overlap with those of the Sir2/SirT1 subfamily [9], [10], [11], [12]. However, *S. cerevisiae* lacks a member of the SirT6/7 subfamily. Other sirtuins are localized in the cytoplasm or mitochondria, where they deacetylate metabolic enzymes and other proteins.

The varied biological functions of sirtuins illustrate the diversity that can be achieved through gene duplication. One interesting observation is that the biological functions of sirtuins have shifted over evolutionary time. For example, the budding yeast ScSir2 and the fission yeast SpSir2 are associated with subtelomeric heterochromatin [13], [14], but their mammalian ortholog SirT1 is not a major driver of subtelomeric chromatin structure. Instead, a different mammalian sirtuin, SirT6, is associated with subtelomeric heterochromatin [12]. Similarly, the roles of sirtuins in the nucleolus have shifted. Budding yeast ScSir2 localizes to the nucleolus [15], where it suppresses unequal recombination of the tandemly repeated rRNA genes [16], [17]. In contrast, the mammalian SirT1 displays little nucleolar localization [18] although it does repress rRNA transcription through several mechanisms [19], [20], [21]. Another sirtuin, SirT7, is predominantly nucleolar in localization [18] and acts in opposition to SirT1 by promoting rRNA transcription [9], [10]. These distinct nucleolar roles of ScSir2, SirT1 and SirT7 indicate that functions have shifted during the course of evolution. Thus, sirtuins have likely adapted and redistributed functions as genes have been duplicated and lost. These features make the Sir2 family interesting and useful for studying the expansion of a gene family.

To understand how sirtuin functions evolve, we have undertaken a comparative study of Sir2/SirT1 subfamily members in various yeast species [22], [23], [24]. As the founding member of the family, ScSir2 has been extensively studied for its roles in heterochromatin formation and suppression of recombination. ScSir2 acts with other Sir, or silent information regulator, proteins to generate a special chromatin structure that is restrictive to transcription [25]. This chromatin represses the cryptic mating-type cassettes to maintain haploid cell identity. It also occurs in subtelomeric regions of the genome where it serves a structural role and represses subtelomeric genes. For the formation of this repressive chromatin, histone H4-K16 is a particularly important target of the ScSir2 deacetylase [3], [26], and deacetylation of this lysine promotes binding of Sir3 to nucleosomes. A second role of ScSir2 is to suppress unbalanced recombination of the tandemly repeated rRNA genes, minimizing the expansion or contraction of the array [16], [27]. ScSir2 is recruited to the non-transcribed spacer regions of the rDNA repeats through its interaction with ScNet1 in the RENT complex [28], [29]. At these locations, ScSir2 promotes the association of cohesin by repressing transcription from a bidirectional RNA polymerase II promoter whose activity would otherwise displace cohesin. The presence of cohesin keeps sister chromatids aligned, minimizing unequal sister chromatid exchange and intrachromosomal recombination among repeats [27], [30], [31].

*ScSIR2* has a paralog, *ScHST1* (homolog of Sir two), which arose through gene duplication approximately 100 million years ago [32]. ScHst1 also represses transcription. However, instead of silencing genes through a long-range spreading mechanism, ScHst1 represses single genes in a promoter-specific manner [33]. It is recruited to target genes, including middle sporulation and NAD^+^ biosynthetic genes [33], [34], through the DNA-binding protein ScSum1 [35], [36].

The budding yeast *Kluyveromyces lactis* diverged from the *S. cerevisiae* lineage before the duplication of *SIR2/HST1* occurred. Consequently, *K. lactis* has a single ortholog of ScSir2 and ScHst1. KlSir2 shares functions with both ScSir2 and ScHst1 [24], [37], [38], indicating that the ancestral gene that became duplicated likely had multiple functions and that subfunctionalization occurred after duplication. Characterization of the interaction domains for the proteins that recruit Sir2 and Hst1 to chromatin revealed that subfunctionalization occurred through the acquisition of complementary inactivating mutations in these domains [23]. It is less well understood how the ancestral Sir2 acquired its multiple functions.

Sir2 has also been studied in the fission yeast *S. pombe*, which diverged from the *Saccharomycetaceae* budding yeast lineage about one billion years ago. Like ScSir2, SpSir2 is involved in the formation of heterochromatin at the subtelomeric regions and cryptic mating-type cassettes, as well as peri-centromeric domains [13], [39], [40]. However, the consequences of histone deacetylation by Sir2 are different in these two species due to the distinct molecular composition of heterochromatin. In particular, SpSir2 targets H3-K9 for deacetylation, enabling the subsequent methylation of this lysine residue, which is then recognized by the heterochromatin proteins Swi6 and Chp2 [39], [40], [41]. SpSir2 is also associated with the rDNA repeats [41], but promoter-specific repression by SpSir2 has not been documented. Thus, given that Sir2 acts in heterochromatin formation and rDNA maintenance in both *S. pombe* and *S. cerevisiae*, these are likely to be ancient functions of Sir2.

An important question is when Sir2 acquired a role in promoter-specific repression mediated by a Sum1-like complex, as occurs for ScHst1 and KlSir2. Therefore, we sought to characterize the functions of Sir2 homologs in species that diverged from *S. cerevisiae* after *S. pombe* but before *K. lactis*. The “CTG” or *Candida* clade of yeast falls in this position, and the genomes for several species in the clade are sequenced and annotated [42], [43]. Although most experimental work has focused on the opportunistic pathogen *C. albicans*, other species in the clade have garnered attention due to their status as emerging pathogens [42], [44], [45]. *Clavispora lusitaniae*, aka *Candida lusitaniae*, was selected for this study because of its phylogenetic position and experimental tractability. *C. lusitaniae* propagates in the haploid state, has a defined sexual cycle including meiosis, and is amenable to genetic manipulation [46], [47], [48]. We used a combination of genetic, biochemical, and high-throughput sequencing approaches to study the function of the *C. lusitaniae* Sir2 ortholog, called ClHst1. ClHst1 is localized to the non-transcribed spacer regions of the rDNA repeats and deacetylates histones at these loci, indicating that ClHst1 acts at the rDNA repeats. However, we found no evidence that ClHst1 associates with subtelomeric regions or impacts gene expression directly. This surprising observation highlights the plasticity of sirtuin function.

## Results

### Identification of Sir2 family members in *C. lusitaniae*

We detected four genes encoding sirtuins in the sequenced genome of *C. lusitaniae*. BLAST analysis and tree construction revealed that the product of gene *CLUG_01277* is most similar in sequence to ScSir2, ScHst1 and KlSir2 and falls into the Sir2/SirT1 subfamily (Figure S1). None of the other sirtuins from *C. lusitaniae* was associated with this subfamily. *CLUG_04582* clustered with *HST2*/SirT2, *CLUG_04799* fell in the *HST3/4* subfamily found only in fungi, and *CLUG_02628* is related to SirT5. An examination of gene synteny using tools on the CoGe Comparative Genomics website [49] was also consistent with *CLUG_01277* being the ortholog of *ScSIR2* and *KlSIR2* (data not shown). Therefore we focused our studies on *CLUG_01277*.

For reference, we also identified *C. albicans* genes encoding sirtuins. Interestingly, this species possesses two proteins that fall into the Sir2/SirT1 subfamily (Figure S1). Of the two, the product of orf 19.4761, which is annotated as *HST1* in the *Candida* genome database [50], is more similar in sequence to *CLUG_01277*. Indeed, orf 19.4761 and *CLUG_01277* are identified as orthologs by the *Candida* gene order browser [51]. The other *C. albicans* protein in the Sir2/SirT1 subfamily is encoded by orf 19.1992, annotated as *SIR2*. This protein is less similar to *CLUG_01277* and has no identified ortholog in *C. lusitaniae* according to the gene order browser. Therefore, in keeping with the *C. albicans* nomenclature, we refer to *CLUG_01277* as *ClHST1*. However, it is critical to note that the *SIR2* and *HST1* genes from *C. albicans* and *S. cerevisiae* arose through distinct gene duplications, and consequently there is no reason to expect *CaHST1* to be more similar in function to *ScHST1* than to *ScSIR2*.

### ClHst1 associates with the rDNA repeats and causes deacetylation

**Figure 1:**
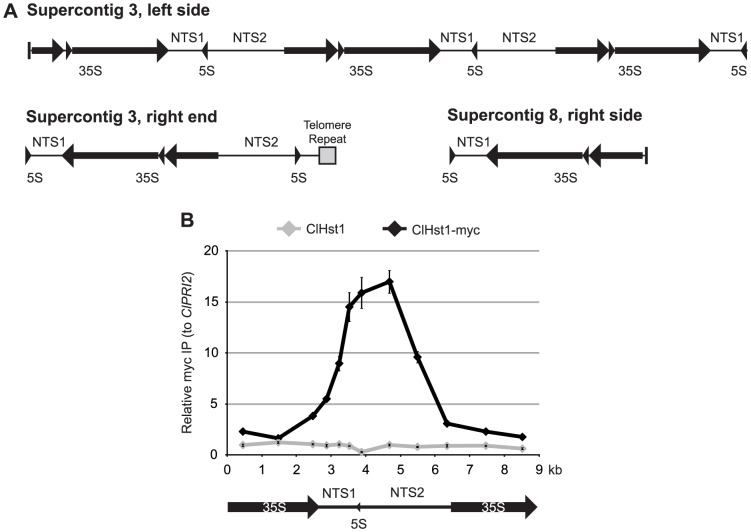
ClHst1 associates with the rDNA repeats. (**A**) Features of the three rDNA loci in *C. lusitaniae* are illustrated. The Pol I-transcribed 35S gene is processed to generate the 18S, 5.8S, and 25S rRNAs (arrows). The Pol III-transcribed 5S gene divides the non-transcribed spacer. All three loci occur near the ends of supercontigs, but only the right end of supercontig 3 has a telomere repeat (gray box), indicating the end of the chromosome. Vertical bars indicate the beginning (3L) or end (8R) of the supercontig sequence. (**B**) The association of ClHst1 across an rDNA repeat was examined by chromatin IP. Anti-myc antibody was used to immunoprecipitate proteins from *ClHST1* (LRY2826) or *ClHST1-MYC* (LRY2858) yeast. The relative enrichment of each probe compared to a control locus, *ClPRI2*, is indicated.

Based on the properties of Sir2/Hst1 in *S. cerevisiae*, *K. lactis*, and *S. pombe*, it is expected that ClHst1 associates with the genomic loci at which it acts. Candidate loci include the rDNA repeats, subtelomeric regions, and promoters of repressed genes. *C. lusitaniae* lacks cryptic mating-type loci, which are sites of Sir2-mediated silencing in other species. To examine the candidate loci, the association of myc-tagged ClHst1 was examined by chromatin immunoprecipitation (IP). We first examined the repeated rRNA genes, which are annotated in three locations in the sequenced genome of *C. lusitaniae*. Three successive repeats are found on the left end of supercontig 3, one repeat with an additional 5S gene occurs on the right end of supercontig 3, and one repeat lacking a non-transcribed spacer is on the right end of supercontig 8 (Figure 1A). It is expected that the actual number of repeats at these loci is much greater, as *S. cerevisiae* encodes 100–200 tandem rRNA genes. Alignment of the three loci revealed significant variation in the non-transcribed spacers, NTS1 and NTS2. Because the rRNA genes at supercontig 3L are repeated in the assembly and display similarity among the non-transcribed spacers, this locus was selected as the template for primer design.

In *S. cerevisiae*, ScSir2 displays peaks of enrichment over the replication fork barrier in NTS1 and the polymerase I promoter in NTS2 [28]. Similarly, in *C. lusitaniae* we observed ClHst1-myc enriched across NTS1 and NTS2 and less abundant over the transcribed region (Figure 1B). The similar distributions of ClHst1 and ScSir2 across an rDNA repeat are consistent with a conserved role for these proteins in shaping chromatin structure at this locus.

**Figure 2:**
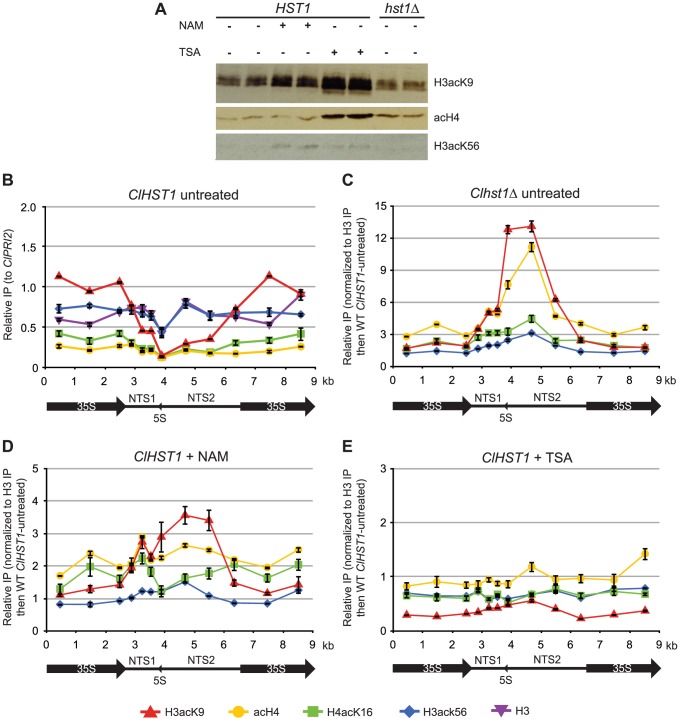
ClHst1 causes deacetylation at the rDNA repeats. (**A**) The relative abundance of acetylated histones was assessed by immunoblotting. Whole-cell lysates were prepared from replicate samples of *ClHST1* yeast (LRY2544) untreated or treated with 25 mM nicotinamide (NAM) or 10 µM trichostatin A (TSA) and from *Clhst1Δ* yeast (LRY2671). Proteins were electrophoretically separated, transferred to membranes, and probed with antibodies against tetra-acetylated H4, H3K9Ac, or H3K56Ac. (**B**) The acetylation of histones H3 and H4 across the rDNA repeat was examined by chromatin IP. Total H3, tetra-acetylated H4, H4-K16Ac, H3-K9Ac, or H3-K56Ac was immunoprecipitated from *ClHST1* (LRY2544) yeast. For each immunoprecipitation, the recovery of probes was normalized to the control locus, *ClPRI2.* (**C–E**) The change in acetylation in deacetylase-deficient yeast relative to wild-type untreated yeast was determined. H3-K9Ac, tetra-acetylated H4, H4-K16Ac, or H3-K56Ac was immunoprecipitated from yeast. For each probe, the enrichment was determined relative to that of total H3. This value was then compared to wild-type untreated cells. Yeast strains used were (**C**) *Clhst1Δ* (LRY2671), (**D**) *ClHST1* (LRY2544) treated with 25 mM nicotinamide (NAM), or (**E**) *ClHST1* (LRY2544) treated with 10 µM trichostatin A (TSA).

The presence of ClHst1 at the rDNA locus is expected to result in deacetylation of histones. Therefore, we compared the levels of histone acetylation in wild-type and *hst1Δ* cells, as well as in cells treated with deacetylase inhibitors. Nicotinamide inhibits sirtuin deacetylases such as ClHst1, and trichostatin A inhibits non-sirtuin deacetylases. If ClHst1 causes histone deacetylation, the level of acetylation within the rDNA array should be greater in *hst1Δ* and nicotinamide-treated cells compared to untreated wild-type cells. The efficacy of the inhibitors at their experimental concentrations was assessed by immunoblotting whole-cell lysates with antibodies against acetylated histones. An increase in acetylation of H3K56 was observed upon treatment with nicotinamide (Figure 2A), presumably due to inactivation of Hst3/4, the orthologs of which target histone H3-K56 globally in *S. cerevisiae* [52], [53]. An increase in H3-K9 and H4 acetylation was observed upon treatment with trichostatin A (Figure 2A). Therefore, the inhibitors did enter the cells and act upon deacetylases.

The distribution of acetylation marks on histones was examined across the rDNA repeat by chromatin IP using antibodies specific for acetylation of histone H3-K9 or K56, histone H4-K16, or tetra acetylated histone H4 (K5,8,12,16). In wild-type cells, most acetylation marks were fairly constant across the repeat (Figure 2B). However, H3K9 acetylation was noticeably lower over NTS1 and NTS2, the same region in which ClHst1 is enriched. We then compared the levels of acetylation across the rDNA locus in wild-type and *hst1Δ* cells and observed increases in H3-K9 acetylation and H4-tetraacetylation across NTS1 and NTS2 in *hst1Δ* cells (Figure 2C). Treatment with nicotinamide had a modest effect upon H3-K9 acetylation, but little impact on other lysines (Figure 2D). In contrast, treatment with TSA had little effect on acetylation (Figure 2E). These results are consistent with ClHst1 deacetylating histones in the non-transcribed spacers and being particularly active towards H3K9 acetylation.

### ClHst1 acts with ClNet1 at the rDNA repeats

Previously studied deacetylases from the Sir2/SirT1 subfamily do not bind directly to DNA. Rather, they are targeted to particular genomic loci through other proteins. For example, ScSir2 is recruited to the rDNA repeats as part of the RENT complex containing the Sir2-interacting protein ScNet1 [29]. However, BLAST analysis of the *C. lusitaniae* genome failed to identify orthologs of known Sir2 interacting proteins from other yeast species. Therefore, we employed mass spectrometry to identify the interacting partners of ClHst1. Myc-tagged ClHst1 was partially purified from a clarified lysate using anti-c-myc agarose resin under conditions that should permit retention of associated proteins. A parallel purification from an untagged strain served as a negative control. Protein samples were separated on an SDS-PAGE gel, and successive gel slices were analyzed by LC-MS/MS. The resulting spectra were searched against the NCBI database to identify proteins. From this analysis, 18 proteins from *C. lusitaniae* were identified as potential interactors with ClHst1 based on their increased enrichment in the myc-tagged ClHst1 sample compared to the untagged ClHst1 strain (Froyd and Rusche, unpublished data). Importantly, one of the identified proteins, the product of *CLUG_02406*, has similarity to ScNet1. We had not initially identified *CLUG_02406* as the ortholog of *ScNET1* because the BLAST score is low and only a small segment of the proteins align. However, both proteins return one another as top BLAST hits.

**Figure 3:**
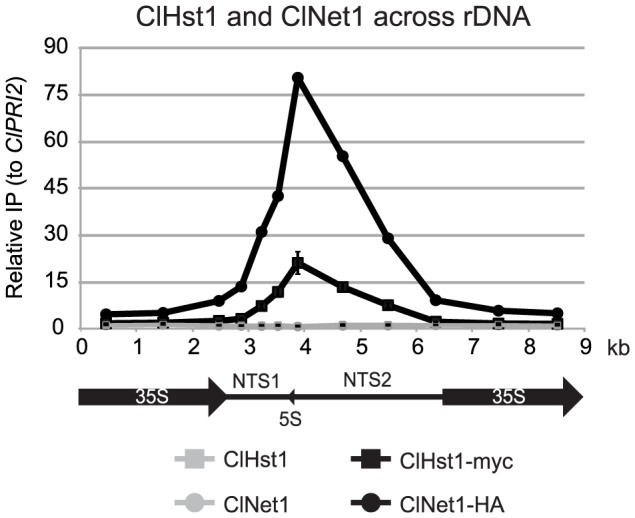
ClHst1 co-localizes with ClNet1 at the rDNA repeats. The association of ClNet1 and ClHst1 across an rDNA repeat was examined by chromatin IP. Anti-HA and anti-myc antibodies were used to immunoprecipitate proteins from *ClHST1 ClNET1* (LRY2826) or *ClHST1-MYC ClNET1-HA* (LRY2917) yeast. The relative enrichment of each probe compared to the control locus, *ClPRI2*, is indicated.

If *CLUG_02406* is indeed the ortholog of *ScNET1*, the protein should associate with the rDNA repeats. We therefore generated an HA-tagged allele of this protein and assessed by chromatin IP whether it associates with the non-transcribed spacer. ClNet1-HA and ClHst1-myc were separately precipitated from a doubly tagged strain and displayed similar distributions across the rDNA repeat (Figure 3). The enrichment of ClNet1 was likely higher than that of ClHst1 because it is closer to the DNA and more efficiently crosslinked. The similar distributions of the two proteins are consistent with both being recruited to the repeat as part of the same complex, as described for the RENT complex in *S. cerevisiae*. These results imply that ClHst1 and ScSir2 act by similar mechanisms to shape chromatin structure at the rDNA locus.

### ClHst1 was not associated with telomeres

An evolutionarily conserved role for ClHst1 in regulating the chromatin structure of the tandem rDNA array is indicated by the co-localization of ClHst1 and ClNet1 at the rDNA locus and by the ClHst1-dependent deacetylation of histones in this region. To determine whether ClHst1 also has a conserved function at subtelomeric regions, we investigated the association of ClHst1 with these loci. Telomeres were identified by BLAST using the previously identified *C. lusitaniae* telomere repeat sequence AAAGAACATCAGTACCTCCCT [42]. This sequence returned hits at six different supercontig ends: 2L, 3R, 4R, 5L, 7L, and 7R. In addition, one hit occurred at an internal location, which is likely the telomerase RNA gene.

**Figure 4:**
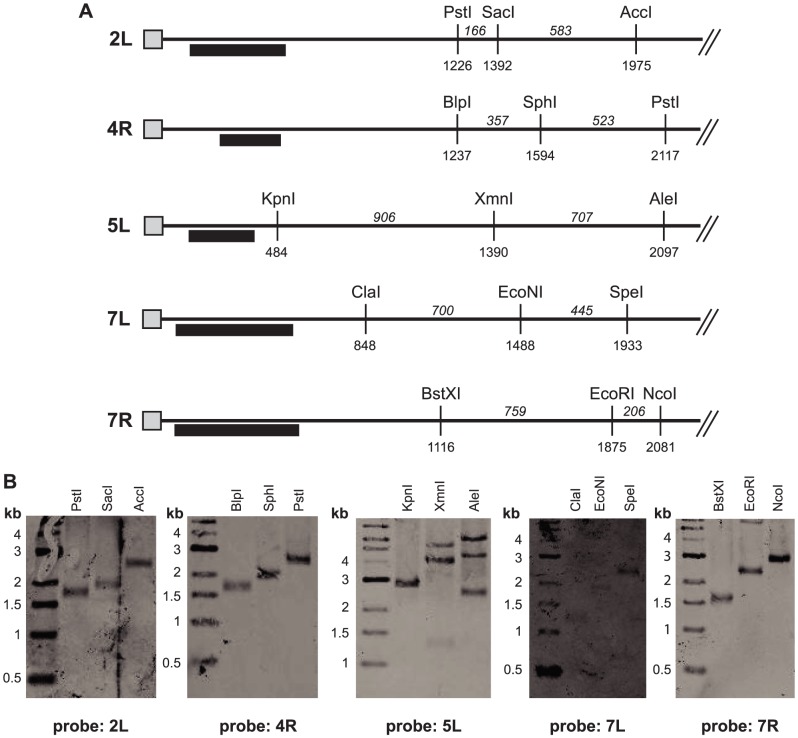
Telomere repeats are located at ends of chromosomes. (**A**) Features at the ends of five supercontigs are illustrated. Telomere repeats are represented by gray boxes. Restriction sites are marked with their distance from the telomere repeat, and the distance between sites is indicated in italics. Probes used for Southern analysis are shown as black rectangles. (**B**) Digested genomic DNA (LRY2826) was separated electrophoretically, transferred to membranes, and hybridized with the indicated probes located near telomere repeat sequences.

To confirm that the identified telomere repeat sequences are indeed located at the ends of chromosomes, Southern blot analysis was performed. Genomic DNA was digested separately with three restriction enzymes that cut 0.5–2.5 kb from each telomere repeat sequence (Figure 4A). The cut DNA was separated electrophoretically, transferred to membranes, and analyzed using probes located just inside the telomere repeat. If the identified sequences are indeed at the ends of the chromosomes, the three digestions should yield bands whose sizes differ by the distances between the restriction sites. In contrast, if the repeats are actually internal sequences and additional unknown sequence occurs beyond the repeats, the sizes of the restriction products will be inconsistent with the prediction. For telomere repeats 2L, 4R, 7L, and 7R, the observed restriction fragments were consistent with the repeat sequence being at the end of the chromosome (Figure 4B). In all four cases, the telomere repeat length can be deduced to be 500–600 bp. Putative telomere 3R was not examined because it is associated with an rDNA array (Figure 1A). For contig 5L, multiple bands were observed, some of which differed in length by the expected amounts. However, these restriction fragments were longer than expected, suggesting that there is approximately 500 bp of additional unknown sequence between the restriction sites and the chromosome end. Nevertheless, four *bona fide* telomeres were identified.

**Figure 5:**
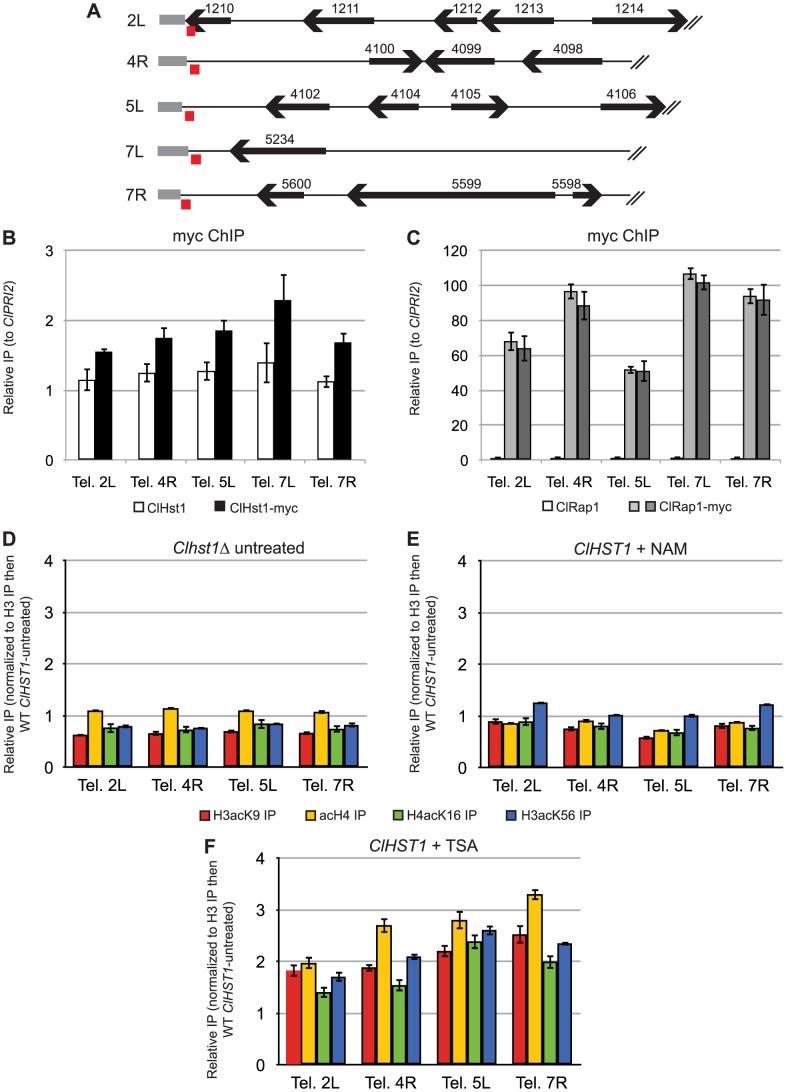
ClHst1 is not associated with telomeres. (**A**) Diagrams represent the genetic features of 10 kbp at ends of contigs bearing telomere repeat sequences. The positions of genes (black arrows), telomere repeat sequences (gray boxes), and PCR products used for chromatin IP analysis (red boxes) are shown. Two dubious ORFs have been omitted. *CLUG_04101* overlaps the telomere repeat sequence of contig 5L, and *CLUG_04103* is antisense to *CLUG_4102*. (**B**) The association of ClHst1 with telomeres was examined by chromatin IP. Anti-myc antibody was used to immunoprecipitate proteins from *ClHST1* (LRY2826) or *ClHST1-MYC* (LRY2858) yeast. The relative enrichment of each probe compared to the control locus, *ClPRI2*, is indicated. (**C**) The association of ClRap1 with telomeres was examined by chromatin IP. Anti-myc antibody was used to immunoprecipitate proteins from *ClRAP1* (LRY2826) or *ClRAP1-MYC* (LRY2859, LRY2860) yeast. The relative enrichment of each probe compared to the control locus, *ClPRI2*, is indicated. (**D–F**) The change in acetylation of histones H3 and H4 at the telomeres was examined in deacetylase-deficient yeast relative to wild-type. Tetra-acetylated H4, H4-K16Ac, H3-K9Ac, or H3-K56Ac was immunoprecipitated from yeast. For each immunoprecipitation, the enrichment was determined relative to that of total H3, and this value was compared to the value for wild-type untreated cells. Yeast strains used were (**D**) *Clhst1Δ* (LRY2671), (**E**) *ClHST1* (LRY2544) treated with 25 mM nicotinamide (NAM), or (**F**) *ClHST1* (LRY2544) treated with 10 µM trichostatin A (TSA).

The same chromatin IP samples that were analyzed for association of ClHst1 with the rDNA repeat (Figure 1B) were examined to determine whether ClHst1 also associates with telomeres. For this analysis, primers were designed to amplify the unique sequences immediately adjacent to the telomere repeats (Figure 5A). Telomere 3R was excluded because it contains an rDNA repeat, which is expected to associate with ClHst1. Surprisingly, ClHst1-myc showed no enrichment at any of the examined telomeres (Figure 5B), implying that ClHst1 is not associated with subtelomeric chromatin. To determine whether the loci being examined had other telomere-like properties, we determined whether they associated with the telomere-binding protein Rap1. Rap1 has multiple functions in *S. cerevisiae*, including recruiting the SIR complex and other proteins to the telomere to regulate chromatin structure and telomere length [54], [55], [56], [57]. Rap1 also associates with telomeres in other species, including *K. lactis*, *C. albicans*, *S. pombe*, and humans [58], [59], [60], [61]. Indeed, we found that ClRap1-myc was enriched 50 to 100-fold at all five queried telomeres (Figure 5C), supporting the conclusion that the telomeres have been properly identified. Moreover, the presence of ClRap1, whose *S. cerevisiae* homolog recruits the SIR complex to telomeres, indicates that these are loci that would be associated with ClHst1 if its mode of recruitment resembled that of ScSir2.

As an alternate approach to detect ClHst1 at telomeres, the level of histone acetylation at these loci was compared in wild-type and *Clhst1Δ* cells. If ClHst1 is present even transiently at telomeres and deacetylates histones, acetylation should increase in *Clhst1Δ* cells. However, no change in acetylation was observed upon deletion of *ClHST1* (Figure 5D). In contrast, when these same samples were analyzed with rDNA-specific primers, histone acetylation levels increased significantly across the non-transcribed spacer in *Clhst1Δ* cells (Figure 2C). These results suggest that ClHst1 is not a major deacetylase at the *C. lusitaniae* telomeres.

The lack of ClHst1 at the telomeres raised the question of whether another deacetylase acts in the subtelomeric regions. Therefore, the level of histone acetylation at these loci was compared in wild-type and deacetylase-deficient cells, as described above for the rDNA locus (Figure 2D, E). If an inhibitable deacetylase is present at the telomeres, acetylation will increase upon treatment with the appropriate inhibitor. Although no change in acetylation was observed upon nicotinamide treatment (Figure 5E), acetylation did increase slightly upon TSA treatment (Figure 5F). Thus, non-sirtuin deacetylases may play a role in telomere structure in *C. lusitaniae*.

### The loss of ClHst1 did not alter expression of most subtelomeric genes

**Figure 6:**
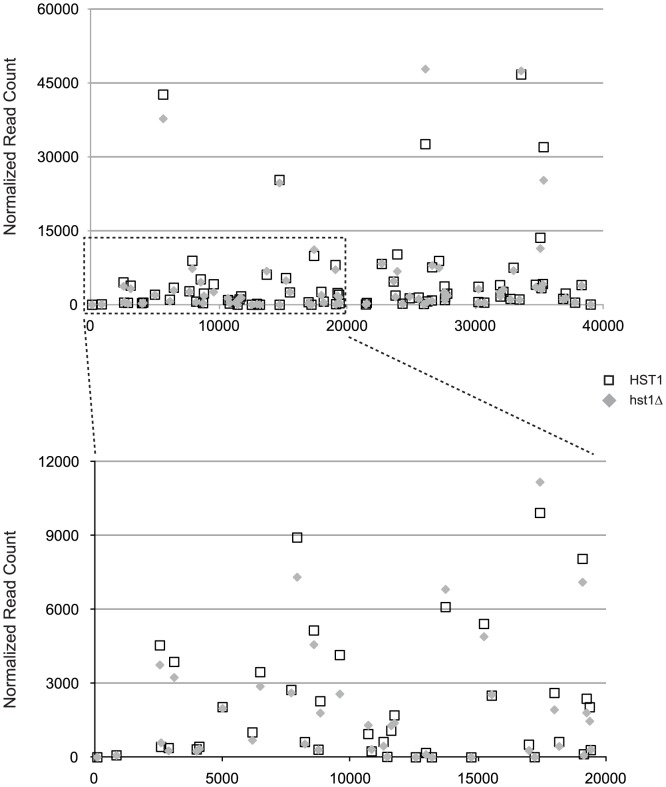
Expression of subtelomeric genes did not change in the absence of ClHst1. Average normalized read counts are plotted for all genes within 40,000 base pairs of a telomere repeat sequence. These genes are located on contigs 2L, 4R, 5L, 7L, and 7R. For each gene, the distance of the start codon from the telomere repeat sequence is plotted on the x-axis. The dashed rectangle in the upper panel is expanded in the lower panel. Strains examined were wild-type (LRY2544; open boxes) and *hst1Δ* (LRY2623 and LRY2671; shaded diamonds).

Unlike ClHst1, Sir2 orthologs in *S. cerevisiae* and *K. lactis* are associated with telomeres, and consequently subtelomeric genes are induced in the absence of these proteins. To confirm the absence of ClHst1 at telomeres, we compared the expression of subtelomeric genes in wild-type and *hst1Δ C. lusitaniae* cells. If ClHst1 is indeed absent from the telomeres, no change in expression of subtelomeric genes is expected. RNA was isolated from two independently constructed *Clhst1Δ* strains as well as the parental wild-type *ClHST1* strain, and cDNA was prepared for Illumina sequencing. The normalized number of reads derived from each gene was compared in *Clhst1Δ* and wild-type strains. All genes within 40 kbp of a telomere repeat sequence were examined, and none were found to differ by more than two-fold (Figure 6). Of the seven genes located elsewhere that were induced, *CLUG_02300* is 2 kbp from the end of a supercontig lacking telomere repeats, and *CLUG_01197* is 24 kbp from an end. However, neither gene displayed properties consistent with being silenced by block of Hst1-mediated heterochromatin. *CLUG_01197* was well-expressed even in the presence of ClHst1, whereas *CLUG_02300* remained poorly expressed in the absence of ClHst1 (Figure S2). Moreover, neighboring genes did not share the expression patterns of these two genes. Therefore, the gene expression data are consistent with the absence of ClHst1 at telomeres.

### ClHst1 has a limited and likely indirect impact on gene expression

**Figure 7:**
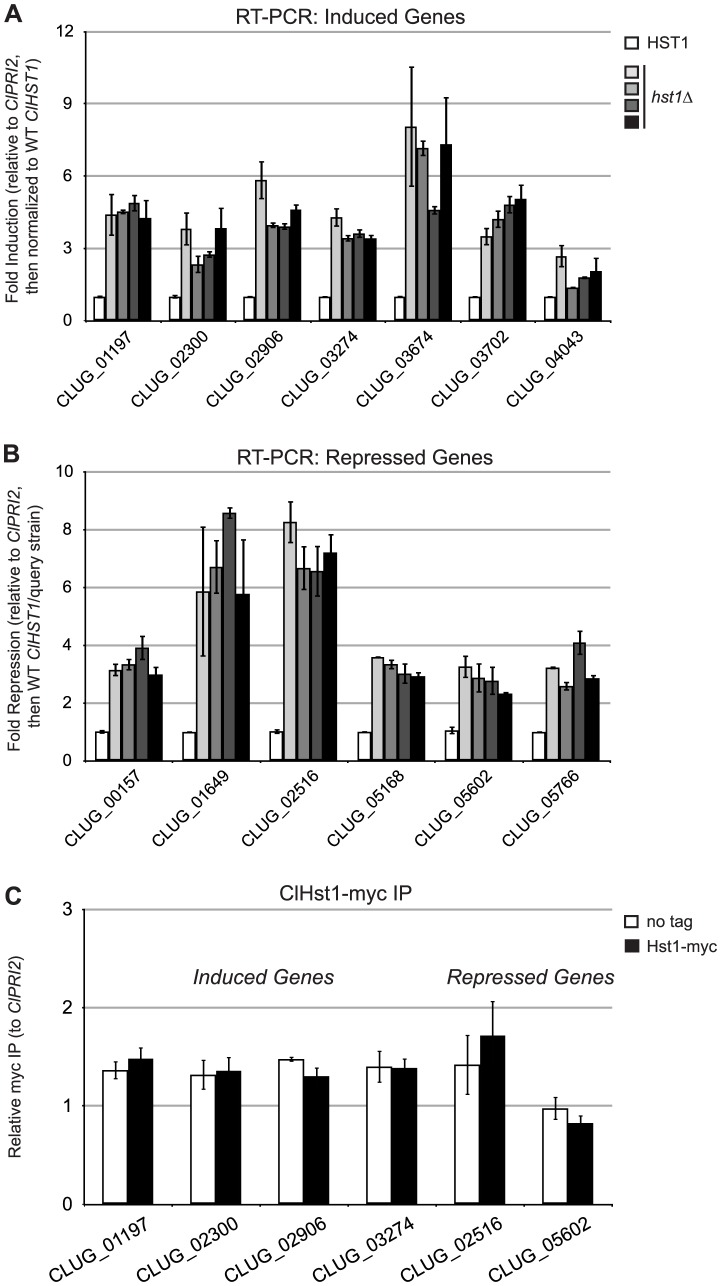
ClHst1 has a limited and indirect impact on gene expression. (**A**) Expression levels were assessed for genes identified by RNA-seq as induced in *Clhst1Δ* strains. Expression was assessed by quantitative RT-PCR in *ClHST1* (LRY2544, white) and four independently constructed *Clhst1Δ* strains (LRY2623, light gray; LRY2671; medium gray; LRY2672, dark gray; LRY2673, black). Levels of mRNA for each gene were first normalized to *ClPRI2* (*CLUG_00368*) and then expressed relative to the WT *ClHST1* strain. (**B**) Expression of genes repressed in *hst1Δ* strains was measured as in part A. For fold repression, the inverse of the fold expression was calculated. (**C**) The association of ClHst1 with promoters of candidate genes was examined by chromatin IP. Anti-myc antibody was used to immunoprecipitate proteins from *ClHST1* (LRY2826) or *ClHST1-MYC* (LRY2858) yeast.

Outside of roles at the rDNA array and subtelomeric loci, ScHst1 and KlSir2 repress transcription of particular genes through a promoter-specific mechanism. Consequently, deletion of *ScHST1* or *KlSIR2* results in the induction of scores of genes [[24], [35], [62]; Hasan and Rusche, unpublished]. To investigate the possibility that ClHst1 has a similar function and to determine in an unbiased way which genes are targets of ClHst1, the RNA-seq data described above were used to identify genes whose expression changed 3-fold or more when *ClHST1* was deleted. These genes were re-analyzed by quantitative reverse transcriptase PCR (Figures 7A and B). A total of 13 genes changed upon the deletion of *ClHST1* and are summarized in Table 1. This number was unexpectedly low, given the number of genes regulated by ScSir2, ScHst1, and KlSir2. In addition, the expression of some of these genes was inconsistent in subsequent RT-PCR analyses (Figure S3), suggesting they are not direct targets of ClHst1. Moreover, although seven of the genes were induced in the absence of ClHst1, as expected for targets of a transcriptional repressor, the remaining six genes were repressed. The functions of the genes were also surprising. ScHst1 and KlSir2 repress genes such as the middle sporulation genes and NAD^+^ biosynthetic genes [24], [33], [34]. In contrast, genes affected by ClHst1 are related to flocculation or adhesion, cell wall biogenesis, and membrane transport. Some genes are also involved in stress response.

**Table 1:** Genes whose expression changed in *hst1Δ* compared to wild-type strains.

	*C. lusitaniae* Gene	RT-PCR	normalized read count		kbp from contig end	*S. cerevisiae* Homolog	*C. albicans* Homolog	Function in *S. cerevisiae* or *C. albicans*
		fold change	wt	*hst1*				
**INDUCED**	*CLUG_01197*	4.5	6403	17414	23.8	*MUC1/CCW12*	*PGA6/YWP1*	GPI-anchored cell surface glycoprotein involved in adhesion
	*CLUG_02300*	3.2	46	124	2.1	*FLO9/5/1*		Lectin-like cell wall protein that binds mannose, involved in flocculation
	*CLUG_02906*	4.6	626	2379	646.5	*FLO5/9/1*		Lectin-like cell wall protein that binds mannose, involved in flocculation
	*CLUG_03274*	3.7	4213	11457	166.2	*SAG1/MUC1*	*ALS4/ALS2*	GPI-anchored cell surface glycoprotein involved in adhesion
	*CLUG_03674*	6.8	1049	3554	848.4	*BIO2*	*BIO2*	Biotin synthase, catalyzes the conversion of dethiobiotin to biotin
	*CLUG_03702*	4.4	13	39	798.1	*STP1/STP2*	*orf19.3643*	Transcription factor activated by proteolysis, activates amino acid permease genes
	*CLUG_04043*	2	28	108	120.0	*YMR244W*	*orf19.5896*	SUN family protein of unknown function
**REPRESSED**	*CLUG_00157*	3.4	1065	202	300.5		*HSP21*	Small heat shock protein (sHSP) with chaperone activity
	*CLUG_01649*	6.7	1871	596	865.3	*PUT1*	*PUT1*	Proline oxidase involved in utilization of proline as sole nitrogen source
	*CLUG_02516*	7.2	336	90	409.8	*VBA5/AZR1/VBA2*	*orf19.7554*	Transporter of the major facilitator superfamily
	*CLUG_05168*	3.2	489	73	127.6	*ESBP6*	*orf19.4337*	Transporter of the major facilitator superfamily
	*CLUG_05602*	2.8	22	4	13.2			unknown
	*CLUG_05766*	3.2	9645	2323	362.2	*CTA1*	*CAT1*	Catalase, breaks down hydrogen peroxide

For each gene, the difference in expression between wild-type and *hst1* cells is provided, based on both RT-PCR and RNA-seq experiments. In addition, the table includes the distance of the start codon from the end of the supercontig and a description of homologous genes in *S. cerevisiae* and *C. albicans*.

To determine whether ClHst1 directly influences the transcription of these genes, chromatin IP was used to assess whether ClHst1 was enriched at their promoters (Figure 7C). However, none of the six examined promoters was enriched for ClHst1. This analysis included the three genes closest to chromosome ends, and the lack of ClHst1 association with these genes is consistent with the absence of ClHst1 near telomere repeats. It was theoretically possible that the myc tag disrupts the interaction of ClHst1 with a protein that targets Hst1 to the gene promoters. If this technical artifact were responsible for the lack of ClHst1-myc association with induced promoters, the expression of these genes would be altered in an *HST1*-myc strain as in *hst1Δ* strains. However, RT-PCR analysis revealed that the pattern of gene expression in the *HST1*-myc cells matched that of wild-type and not *hst1Δ* cells (Figure S3). Therefore, Hst1-myc must be functional. Thus, ClHst1 does not directly regulate the transcription of the genes whose expression changed in its absence, and ClHst1 is unlikely to repress transcription through a promoter-specific mechanism.

## Discussion

### Functions of ClHst1

This study demonstrates that the single member of the Sir2/SirT1 subfamily in *C. lusitaniae*, ClHst1, possesses only a subset of the activities observed for its orthologs in other yeast species. ClHst1 has retained the ability to associate with the non-transcribed portions of rDNA repeats, where it causes deacetylation. It is likely that ClHst1 acts through a similar mechanism as ScSir2 to regulate chromatin structure and hence repress unbalanced recombination within the tandem rDNA array. These findings support the widely held notion that homologous proteins have similar functions from species to species.

In contrast, no evidence was obtained supporting a role for ClHst1 in the subtelomeric regions of *C. lusitaniae*. Although the genome of this species has not been as extensively annotated as the genomes of other yeast, we believe we have identified *bona fide* telomeres. First, the telomere repeat sequence is found primarily at the ends of supercontigs, where it is reiterated 16–24 times, as would be expected for a telomere. The only exception is the likely gene encoding telomerase RNA, for which 1.3 units of the repeat occur at an internal location. Thus, there is no evidence that internal tracts of telomere repeat sequence are found in *C. lusitaniae*. Second, Southern blot analysis is consistent with the identified tracts of telomere repeats being at the ends of chromosomes. Finally, the telomere binding protein Rap1 is associated with the presumed telomeres, as expected. Nevertheless, our observations are inconsistent with ClHst1 being associated with these telomeres. Subtelomeric DNA was not enriched in ClHst1-myc chromatin IP samples that did show enrichment of rDNA. In addition, no change in acetylation of subtelomeric histones was observed upon deletion of *ClHST1* or treatment with the deacetylase inhibitor nicotinamide, although both conditions resulted in changes in histone acetylation at the rDNA repeats. Moreover, the expression of subtelomeric genes did not change upon deletion of *ClHST1*. Thus, ClHst1 appears to lack the ability to promote heterochromatin formation and silencing of subtelomeric genes. This finding is surprising given the presumed importance of maintaining proper telomere structure. Moreover, these results highlight the limitations of extrapolating the function of a protein from orthologous proteins in other species.

ClHst1 was also found not to have an impact on gene expression through a promoter-specific mechanism, as has been observed for KlSir2 and ScHst1. Deletion of *ClHST1* caused few changes in gene expression, and ClHst1 was not present at the promoters of those genes whose expression did change. Therefore, ClHst1 does not appear to be directly responsible for their regulation. Instead, deleting *ClHST1* may cause mild stress, which in turn alters gene expression. Indeed many of the genes whose expression does change respond to stress in *C. albicans*. Alternatively, ClHst1 may deacetylate other proteins that regulate these genes.

Our data also provide insight into the *in vivo* substrates of the ClHst1 deacetylase. Of the histone modifications examined, acetylation of H3-K9 was most affected by the absence of ClHst1. H3-K9 is also a documented substrate of Sir2 in *S. pombe*. A significant increase in H4 acetylation at the rDNA non-transcribed spacer was also detected using an antibody against tetra-acetylated H4. In addition, acetylation of total cellular H4 increased in *hst1Δ* compared to wild-type cells (Figure 2A). Thus, ClHst1 must deacetylate at least one of the lysines in the N-terminus of H4. H4-K16 is a key target of Sir2 in *S. cerevisiae*, but only modest changes in acetylation of this lysine were detected using a specific antibody.

### Duplication and loss of SIR2 genes in yeast species

**Figure 8:**
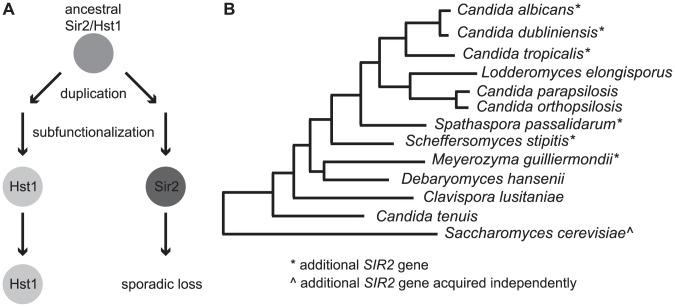
The ancestral SIR2 gene may have duplicated and subfunctionalized prior to loss of one paralog. (**A**) A model is shown depicting the hypothesized evolutionary history of the *SIR2* gene within the CTG clade. An early duplication followed by subfunctionalization partitioned the ancestral functions. The product of one gene (*HST1*) acted at the rDNA locus, whereas the other (*SIR2*) possessed different functions, perhaps including a role at telomeres. In some lineages, including *C. lusitaniae*, only the *HST1* gene was retained. (**B**) A species tree indicates with an asterisk (*) organisms whose genomes encode the additional *SIR2* gene. The maximum likelihood tree is based on [43].

The observation that ClHst1 performs only a subset of the activities possessed by its *Saccharomyces* orthologs raises the question of how this loss occurred. One possibility (Figure 8A) is a multi-step process, in which duplication and subfunctionalization led to the partitioning of functions between two Sir2 paralogs, one of which was subsequently lost. This model is consistent with the observation that other species of the CTG (*Candida*) clade, including *C. albicans*, have a second gene from the Sir2/SirT1 subfamily (Figures 8B, S4). This gene is annotated as *SIR2* in *C. albicans*. Phylogenetic analysis of Sir2 and Hst1 proteins from CTG clade species reveals that these two paralogs cluster separately (Figure S4), consistent with a duplication having occurred in a common ancestor of these species. The sporadic presence of *SIR2* throughout the clade (Figure 8B), suggests that *SIR2* was lost independently in several lineages. Prior to these losses, the duplicated genes would have subfunctionalized, such that *HST1* retained the capacity to regulate rDNA whereas *SIR2* had other functions, perhaps including the generation of subtelomeric chromatin. It is noteworthy that *SIR2* rather than *HST1* has been lost in all *Candida* lineages bearing a single S*IR2/HST1* gene, implying that *HST1* is more critical for survival.

Although phylogenetic analysis suggests that the duplication of the *Candida SIR2* and *HST1* genes occurred in a common ancestor of the CTG clade species, it is unclear exactly when this event happened. Duplication at the base of the CTG clade is consistent with the absence of genes similar to *CaSIR2* in non-CTG clade species. However, an earlier timing for the duplication is suggested by the clustering of CaHst1 with *Saccharomyces* Sir2 and Hst1 and not CaSir2 (Figure S1).

The loss of sirtuin genes has occurred repeatedly during the course of evolution, as particular lineages often lack one or another subfamily. It is also likely that subsequent to these losses, other sirtuins have acquired compensatory functions. Consequently, the biological functions of sirtuins shift from species to species. In humans, SIRT6 and SIRT7 have functions related to those of yeast Sir2, although they are not orthologous. In particular, SIRT6 is associated with subtelomeric heterochromatin and influences transcription at other loci as well [11], [12]. SIRT7 is associated with the rDNA, although its ability to repress recombination between the rDNA repeats has not been examined [9], [10]. In contrast, SirT1, the ortholog of Sir2, has its major functions away from the telomeres and nucleolus. Given that *S. cerevisiae* and related yeast species do not encode orthologs of either SIRT6 or SIRT7, it is possible that yeast Sir2 acquired functions previously associated with these sirtuins.

This work demonstrates that, although homologous proteins in two species can have similar functions, this is not always the case. It is therefore prudent to acquire experimental support rather than extrapolating function from one species to another.

### Potential absence of heterochromatin in *C. lusitaniae*

The absence of ClHst1 in the subtelomeric regions of *C. lusitaniae* leaves unresolved the nature of chromatin at these loci. In most eukaryotic species, subtelomeric domains are associated with heterochromatin, generated in part by Sir2 family deacetylases. Heterochromatin formation can also be initiated by siRNA-containing RISC-like complexes or by methylation of histone H3-K9. However, neither mechanism is likely in *C. lusitaniae*, given that the genome does not encode either the siRNA-presenting protein Argonaute or the methyltransferase specific for H3-K9. It does remain possible that a deacetylase(s) other than ClHst1 acts at telomeres. Thus, *C. lusitaniae* presents an opportunity to discover how subtelomeric chromatin can be reconfigured.

## Materials and Methods

### Yeast growth and transformation

*Clavispora lusitaniae* strains were grown at 30°C in YPD (1% yeast extract, 2% peptone, 2% glucose). Electroporation conditions were derived from previous protocols [46], [63], [64]. Briefly, 75 OD of cells were harvested at an optical density between 1.5 and 1.8 and resuspended in 10 mL 0.1 M lithium acetate, 1× TE, and 10 mM DTT. Cells were shaken one hour at room temperature, collected, washed in 25 mL cold sterile water, and suspended in 0.5 mL cold 1 M sorbitol. Electroporation reactions were set up in 0.2 mm cuvettes using 40 µL cells, 2 µL 10 mg/mL salmon sperm DNA, and 1–5 µg DNA in a volume no more than 5 µL. Electroporation conditions were 1800 V, 200 Ω, and 25 µF. After electroporation, cells were incubated in YPD four hours at 30°C and then spread on selective media (YPD + 200 µg/ml CloNat or supplemented yeast minimal medium lacking uracil or histidine). Cells to be plated on 5-FOA medium were first incubated overnight at 30°C.

### Strain construction

Strains used in this study were derived from CL143 (Table 2), which is congenic to the sequenced strain ATCC 42720. Genomic sequence was obtained from the *Candida* database hosted by the Broad Institute (http://www.broadinstitute.org/annotation/genome/candida_albicans/MultiHome.html), and orthologs of *S. cerevisiae* and *C. albicans* genes were identified by BLAST analysis (Table 3). To aid in the genetic manipulation of *C. lusitaniae*, genes were first subcloned into *S. cerevisiae* shuttle vectors. Each gene, including 800–1000 bp upstream and downstream of the open reading frame, was amplified by PCR from genomic DNA. The PCR products were then ligated into the shuttle vector pRS415 [65]. These plasmids were subsequently modified to generate deletion constructs, in which the open reading frame was replaced by a selectable marker, or tagging constructs, in which an epitope tag and selectable marker were inserted just after the last non-stop codon of the open reading frame. Finally, the deletion or tagging cassettes were integrated into the *C. lusitaniae* genome through homologous recombination.

**Table 2:** Yeast strains used in this study.

Strain	Genotype	Source
CL143	MAT a chx^r^ (congenic to ATCC 42720)	Reedy and Heitman
LRY2544	CL143 *ura3Δ*	
LRY2623	CL143 *ura3Δ hst1Δ::ClURA3*	
LRY2671	CL143 *ura3Δ hst1Δ::ClURA3*	
LRY2672	CL143 *ura3Δ hst1Δ::ClURA3*	
LRY2673	CL143 *ura3Δ hst1Δ::ClURA3*	
LRY2826	CL143 *ura3Δ his1Δ*::*FRT*	
LRY2858	CL143 *ura3Δ his1Δ::FRT HST1::MYC-ClHIS1*	
LRY2859	CL143 *ura3Δ his1Δ::FRT RAP1:: MYC -ClHIS1*	
LRY2860	CL143 *ura3Δ his1Δ::FRT RAP1:: MYC -ClHIS1*	
LRY2917	CL143 *ura3Δ his1Δ::FRT HST1:: MYC -ClHIS1 NET1::HA-ClURA3*	

The genotypes of yeast strains used in this study are given.

**Table 3:** Genes modified in this study.

Common Name	*C. lusitaniae* Systematic Name	*S. cerevisiae* Homolog	Biological Function in *S. cerevisiae*
*ClHST1*	*CLUG_01277*	*ScSIR2*	Silences *HML*, *HMR*, and telomeres; represses unequal recombination of rDNA repeats
		*ScHST1*	Represses gene expression in promoter-specific manner
*ClNET1*	*CLUG_02406*	*ScNET1*	subunit of the RENT complex; involved in nucleolar silencing and telophase exit
*ClRAP1*	*CLUG_02967*	*ScRAP1*	Telomere maintenance; recruits Sir complex to telomeres; transcriptional regulator
*ClURA3*	*CLUG_04406*	*ScURA3*	Biosynthesis of uracil
*ClHIS1*	*CLUG_02145*	*ScHIS1*	Biosynthesis of histidine

Genes modified in this study are listed with their common and systematic gene names.

Strains auxotrophic for uracil or histidine were generated by deleting the complete open reading frames of *URA3* or *HIS1*. To create the *Clura3Δ* deletion cassette, plasmid pLR787 containing *ClURA3* was amplified by PCR using oligos that bridge and excise the open reading frame to create pLR810. Because loss of *URA3* confers resistance to 5-FOA, no selectable marker replaced *URA3*. To generate the *Clhis1Δ* deletion cassette, the *caSAT1* flipper cassette from pSFS2 [64] was used. This cassette confers resistance to ClonNat and is flanked by *FRT* sites that allow it to be excised from the genome upon induction of a *FLP* recombinase gene located in the cassette. The *caSAT1* flipper cassette was amplified by PCR using primers whose 5′ ends matched sequence immediately upstream and downstream of the *ClHIS1* open reading frame. The amplified cassette was then incorporated into pLR891 in place of the *HIS1* open reading frame by homologous recombination in *S. cerevisiae* to generate pLR909. The resulting *Clhis1Δ::FRT-caFLP-caSAT1-FRT* cassette was then integrated into the genome of *C. lusitaniae*. Before a stable integrant was isolated, the *caSAT1* flipper excised itself, leaving a single *FRT* site in place of the *ClHIS1* gene. We surmise that the maltose-inducible *FLP* gene was expressed at a low level in the absence of maltose.

As another tool, we generated a plasmid containing a myc tag linked to the *ClHIS1* gene because we suspected that the *C. albicans HIS1* gene in the tagging plasmid pFA-MYC-*HIS1* [66] was not expressed in *C. lusitaniae*. The *ClHIS1* gene, including its promoter and downstream sequence, was amplified from pLR891 using primers whose 5′ ends matched sequence flanking *CaHIS1* in pFA-MYC-*HIS1*. The *ClHIS1* PCR product was then incorporated into pFA-MYC-*HIS1* in place of the *CaHIS1* gene by a PCR stitching strategy to create pLR950.

To create the *Clhst1Δ* deletion cassette, the *ClURA3* gene was amplified using primers whose 5′ ends matched sequence immediately upstream and downstream of the *ClHST1* open reading frame. This PCR product was incorporated into pLR786 in place of *ClHST1* by homologous recombination to generate pLR811. To add a myc tag to the C-terminus of *ClHST1*, the myc tag and *ClHIS1* marker were amplified from pLR950. The resulting PCR product was inserted at the 3′ end of the *ClHST1* gene by PCR stitching to generate pLR956. Using a similar strategy, a myc tag was added to the C-terminus of ClRap1, to generate pLR958. To add the HA tag to the C-terminus of *ClNET1*, the HA tag and *CaURA3* marker were amplified from pFA-HA-URA3 [66], and this PCR product was incorporated at the 3′ end of the *ClNET1* gene by PCR stitching to generate pLR963. The *CaURA3* gene and promoter were then replaced with *ClURA3* to generate pLR973. Correct integration of all tags was confirmed by sequencing.

### Phylogenetic analysis of sirtuin genes

Sequences of sirtuin proteins were obtained from Genbank, the yeast gene order browser [32], and the *Candida* gene order browser [43], [51]. Phylogenetic trees of the core catalytic domains were constructed using the CLUSTAL_W algorithm of MEGA version 4.0 [67], and distances were calculated with default parameters of the neighbor-joining algorithm applying amino: poisson correction [68] in a pair-wise deletion procedure. The robustness of tree topologies was evaluated by 500 bootstrap replications.

We considered the possibility that a second *SIR2* gene does exist in *C. lusitaniae* but is not represented in the sequenced genome. We first searched for *SIR2* at its syntenic position. In the closest species that contains *SIR2*, *M. guillermondii*, *SIR2* is in the center of a block of seven genes, the other six of which are arranged in the same order in *C. lusitaniae*. The position where the absent *SIR2* gene should be is 1.1 kb in length and contains no missing sequence. Thus, if *SIR2* is present in *C. lusitaniae*, it must have relocated. To search for a partial, unannotated reading frame for *SIR2*, we performed TBLASTN analysis comparing the *M. guillermondii* Sir2 protein to the *C. lusitaniae* genomic sequence. Four hits were returned, corresponding to the four known sirtuin genes. Thus, the available genomic sequence for *C. lusitaniae* lacks the *SIR2* gene.

### DNA blotting and analysis of telomere repeat locations

To verify the accuracy of the publically available genomic sequence adjacent to *C. lusitaniae* telomere repeats, PCR was performed using sets of overlapping primers spanning 3 kb from the telomere repeats. The size of each PCR product was examined, and, with the exception of telomere 4R, all were consistent with the published sequence. Some PCR products were sequenced to obtain approximately one kb of additional sequence at 4R and small annotated gaps in the sequence for telomeres 2L and 5L. These sequences were deposited in GenBank (telomere 2L: KF015764; telomere 4R: KF015766; telomere 5L: KF015765).

For Southern blotting, 150 µg genomic DNA (LRY2826) was treated with restriction enzymes, electrophoretically separated on 1% agarose gels, and transferred to Biodyne B Nylon membrane in 10× SSC buffer (3 M NaCl, 0.3 M trisodium citrate) by capillary transfer. The membrane was rinsed in 2× SSC, air dried, and crosslinked by exposure to uv light. Probes were generated by PCR using oligonucleotides listed in Table S1. PCR products were purified using a QIAquick PCR purification kit and labeled with Biotin-High Prime (Roche Diagnostics, Cat # 11585649910) according to manufacturer's instructions. Blots were hybridized overnight in 5× SSPE , 10% dextran sulfate, 2% (w/v) SDS, 1× Denhardt's solution, 10 µg/ml denatured salmon sperm DNA at 65°C. Blots were washed twice for five minutes with 2× SSPE at 50°C, twice in 2× SSPE, 1% (w/v) SDS, and twice in 0.1× SSPE for 15 minutes at 60°C. The membrane was blocked in Odyssey Blocking Buffer containing 1% SDS for 30 minutes at room temperature and developed in streptavidin IRDye 800CW solution (1∶10,000 in Blocking Buffer plus 1% SDS) in the dark at room temperature for 30 minutes. The blot was washed 3 times in 1× PBST, 0.1% Tween-20, once in 1× PBS, and scanned using the Odyssey Infrared Imaging System, Licor Biosciences.

### Chromatin immunoprecipitation

Chromatin IPs were performed by harvesting approximately 100 OD of logarithmically growing cells at an OD_600_ of 3.0–4.0. For deacetylase inhibition, overnight cultures were diluted and grown for 6 hours at 30°C with no inhibitor, 25 mM nicotinamide, or 10 µM trichostatin A. Cells were crosslinked in two ways. For histone immunoprecipitations, cells were rocked at room temperature in 1% formaldehyde for 30 minutes. For Hst1-myc, Rap1-myc, and Net1-HA immunoprecipitations, cells were washed twice with PBS, resuspended in DMA (10 mM dimethyl adipimidate, 0.1% DMSO, 1× PBS) and rocked for 45 minutes, washed again in PBS, resuspended in 1% formaldehyde in PBS and rocked for 45 minutes. The preparation of soluble chromatin and immunoprecipitation were performed as previously described [69], except that cells were lysed by vortexing for 45 minutes. Immunoprecipitations were performed using 5–10 µl of α-myc (Millipore 06-549), α-HA (Sigma H6908), α-histone H3 (Upstate 06-755), α-histone H3 acetyl-lysine 9 (Upstate 06-942), α-histone H3 acetyl-lysine 56 (Upstate 07-677), α-tetraacetyl histone H4 (Millipore 06-866), or α-histone H4 acetyl-lysine 16 (Active Motif 39167) antibody. Chromatin IP samples were analyzed by real-time PCR using a standard curve prepared from input DNA and oligonucleotides listed in Table S2. The amounts of the immunoprecipitated DNA at experimental loci and a control locus, *ClPRI2*, were determined relative to the standard curve, and then the relative enrichment of the experimental loci compared to the control locus was calculated. To assess the change in acetylation, the amount of immunoprecipitated DNA in the acetyl IP was normalized to total histone H3, and this value was compared to the value for wild-type untreated cells. Results represent IPs from two independent cultures of each transformed strain, and the SEM was calculated from the differences in the relative enrichment from the mean.

### Analysis of bulk histone acetylation

To assess whether treatment with deacetylase inhibitors was effective, 25 OD equivalents of treated cells were resuspended in 400 µl lysis buffer (50 mM HEPES-KOH, pH 7.5, 0.5 M NaCl, 10% glycerol, 0.5% NP-40, 1 mM EDTA, 10 mM DTT, 1× Complete Protease Inhibitors (Roche), 1 mM PMSF, 1 µg/mL pepstatin A, 2 mM benzamidine) and approximately 0.5 mL 0.5 mm Zirconia/Silica beads (Biospec). Cells were lysed by vortexing 30 minutes at 4°C. Samples were spun 10 minutes at 13,200 rpm at 4°C. Supernatants were collected, combined with 3× protein sample buffer, and heated 5 minutes at 95°. Samples were fractionated on 12% polyacrylamide-SDS gels, transferred to nitrocellulose membranes, and probed with either α-histone H3 acetyl-lysine 9 (Upstate 06-942), α-histone H3 acetyl-lysine 56 (Upstate 07-677), or α-tetraacetyl histone H4 (Millipore 06-866) and detected by chemiluminescence (GE RPN2135).

### Gene expression analysis

RNA was isolated from 20 OD logarithmically growing cells with optical densities between 3 and 4 [70]. DNA was removed from up to 100 µg RNA using the RNase-Free DNase kit (QIAGEN) and further purified using the RNeasy Mini Kit (QIAGEN). To confirm that the DNase treatment was complete, approximately 100 ng of DNase-treated RNA was used in a PCR reaction containing primers for the *ClACT1* transcript.

For RNA-seq analysis, RNA samples were processed for Illumina sequencing by the Duke IGSP sequencing facility. Sequencing reads were mapped to known *C. lusitaniae* open reading frames using BWA [71] , and the normalized number of hits per gene was calculated. To identify genes whose expression changed in *hst1Δ* strains, the normalized numbers of hits were compared for wild-type and *hst1Δ* strains.

For RT-PCR analysis, 1 µg of DNA-free RNA was used for cDNA synthesis as previously described [38]. To quantify the relative amount of mRNA transcripts, cDNA was analyzed by real-time PCR in the presence of SYBR green using a Bio-Rad iCycler. Oligonucleotide sequences are provided in Table S3. A standard curve was generated with genomic DNA isolated from CL145. Transcript levels of queried genes were first normalized to *ClPRI2* (*CLUG_00368*) for each sample. The fold-change was then calculated by normalizing to the strain expressing wild-type *ClHST1*. Results represent the average fold change of two independent cultures. The standard error measurement (SEM) was calculated from the differences in fold induction of two independent cultures from the mean.

### Protein purification and mass spectrometry

To identify proteins associated with ClHst1, the protein was affinity purified from yeast, and co-purifying proteins were identified by mass spectrometry. Six liters of cells were harvested at an OD_600_ around 3. Cells were washed with ice cold water, then ice cold lysis buffer (50 mM HEPES-KOH, pH 7.5, 0.5 M NaCl, 10% glycerol, 0.5% NP-40, 1 mM EDTA, 1 mM PMSF, 1 µg/mL pepstatin A, 2 mM benzamidine, 2 µg/mL leupeptin). Cell pellets were stored at −80°C. For lysis, cell pellets from 3 liters were resuspended in ice cold lysis buffer containing 100 µl Yeast Protease Inhibitor (Sigma P8215) and placed in a 50 mL BeadBeater small chamber assembly (Biospec cat. no. 110803-50SS) containing 0.5 mm glass beads as indicated by the manufacturer. Additional lysis buffer was added to fill the chamber, and cells were homogenized by alternately beating 30 seconds then resting 90 seconds for 45 minutes at 4°C. The lysates from each assembly were split into 2 tubes and rocked with 50 µl 10 mg/mL heparin and 50 µl 2.5 U/mL benzonase per tube for 15 minutes at room temperature. Lysates were centrifuged for 20 minutes at 14,000×g at 4°C. Supernatants were then spun in an ultracentrifuge for 90 minutes at 45,000 rpm at 4°C. After ultracentrifugation, supernatants were combined (two into one 50 mL tube), and protein concentrations were determined using the BioRad assay (BioRad 500-0006).

Lysates containing 580–750 mg of total protein were incubated overnight at 4°C with 600 µl of a 50% slurry of pre-washed anti-c-myc agarose resin (Sigma A7470). The resin was collected and washed once with 10 mL lysis buffer, once with 10 mL PBS+0.5% NP-40 with protease inhibitor cocktail, then twice with 10 mL PBS with protease inhibitor cocktail. The resin was resuspended in 500 µl PBS with protease inhibitor cocktail and transferred to a microcentrifuge tube and collected. The resin was then submitted to the Duke Proteomics Core Facility, where the sample was eluted by Rapigest and run on an SDS-PAGE gel. Bands were selected for isolation by in-gel digestion and LC-MS/MS analysis by FastMS2.

## Supporting Information

Figure S1 Phylogenetic tree of yeast and metazoan sirtuin proteins. A phylogenetic tree represents the relationships of the core catalytic domains of sirtuin proteins from yeast and metazoan species. The tree was constructed using the CLUSTAL_W algorithm of MEGA version 4.0 [67], and distances were calculated with default parameters of the neighbor-joining algorithm applying amino: poisson correction [68] in a pair-wise deletion procedure. The robustness of tree topologies was evaluated by 500 bootstrap replications. All known sirtuins from the following species were included: Scer (*Saccharomyces cerevisiae*), Zrou (*Zygosaccharomyces rouxii*), Klac (*Kluyveromyces lactis*), Calb (*Candida albicans*), Clus (*Clavispora lusitaniae*), Spom (*Schizosaccharomyces pombe*), Hsap (*Homo sapiens*), and Dmel (*Drosophila melanogaster*).
(PDF)

Figure S2 Induced genes near chromosome ends. (**A**) Diagrams display the genetic features surrounding two genes induced in *hst1Δ* cells compared to wild-type cells (indicated with stars). Genes marked with asterisks are dubious because they lack similarity to known proteins in Genbank and are antisense to *bona fide* genes. (**B**) Average normalized read counts derived from RNA-seq experiment are plotted for genes located near the right end of supercontig 2. (**C**) Normalized read counts are plotted for genes located near the right end of supercontig 1.
(PDF)

Figure S3 The expression of Hst1-influenced genes is the same in wild-type and HST1-myc strains. (**A**) Expression was assessed by quantitative RT-PCR in *ClHST1* (LRY2826, white), *ClHST1*-myc (LRY2858, black) and two independently constructed *Clhst1Δ* strains (LRY2671; medium gray; LRY2672, dark gray). Levels of mRNA for each gene were first normalized to *ClPRI2* (*CLUG_00368*) and then expressed relative to the WT *ClHST1* strain. (**B**) Expression of genes repressed in *hst1Δ* strains was measured as in part A. For fold repression, the inverse of the fold expression was calculated.
(PDF)

Figure S4 Phylogenetic tree of Sir2 and Hst1 proteins from CTG clade species. A phylogenetic tree represents the relationships of the core catalytic domains of Sir2 and Hst1 proteins from CTG clade species. The tree was constructed using the CLUSTAL_W algorithm of MEGA version 4.0 [67], and distances were calculated with default parameters of the neighbor-joining algorithm applying amino: poisson correction [68] in a pair-wise deletion procedure. The robustness of tree topologies was evaluated by 500 bootstrap replications. Sir2 and Hst1 from the following species were included: Calb (*Candida albicans*), Cdub (*Candida dubliniensis*), Ctro (*Candida tropicalis*), Spas (*Spathaspora passalidarum*), Ssti (*Schefferomyces stipitis*), Cpar (*Candida parapsilosis*), Cort (*Candida orthopsilosis*), Lelo (*Lodderomyces elongisporus*), Cten (*Candida tenuis*), Dhan (*Debaryomyces hansenii*), Mgui (*Meyerozyma guilliermondii*), and Clus (*Clavispora lusitaniae*).
(PDF)

Table S1 Oligonucleotides used for ChIP. The sequences of oligonucleotides used for chromatin IP experiments are provided.
(PDF)

Table S2 Oligonucleotides used for RT-PCR. The sequences of oligonucleotides used for reverse transcriptase- qPCR analyses are provided.
(PDF)

Table S3 Oligonucleotides used to generate probes. The sequences of oligonucleotides used to generate probes for Southern analysis are provided.
(PDF)
