# Supplementary figures and images for: The Deacetylase Sir2 from the Yeast Clavispora lusitaniae Lacks the Evolutionarily Conserved Capacity to Generate Subtelomeric Heterochromatin
Source: PLoS Genet. 2013 Oct 31;9(10):e1003935. doi: 10.1371/journal.pgen.1003935 (PMC3814328; doi:10.1371/journal.pgen.1003935)

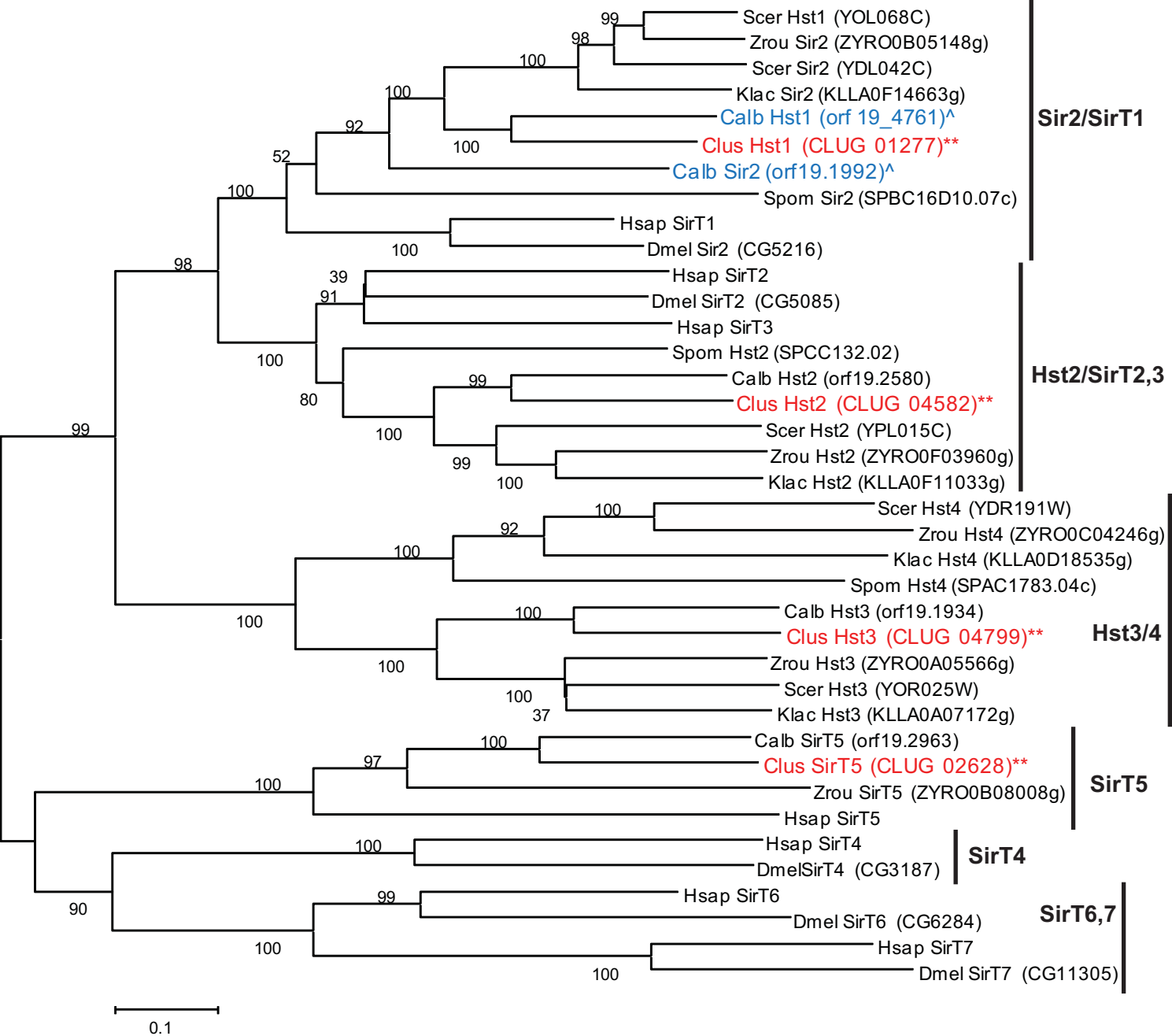

Supplement: Figure S1 — Phylogenetic tree of yeast and metazoan sirtuin proteins. A phylogenetic tree represents the relationships of the core catalytic domains of sirtuin proteins from yeast and metazoan species. The tree was constructed using the CLUSTAL_W algorithm of MEGA version 4.0 [67], and distances were calculated with default parameters of the neighbor-joining algorithm applying amino: poisson correction [68] in a pair-wise deletion procedure. The robustness of tree topologies was evaluated by 500 bootstrap replications. All known sirtuins from the following species were included: Scer (Saccharomyces cerevisiae), Zrou (Zygosaccharomyces rouxii), Klac (Kluyveromyces lactis), Calb (Candida albicans), Clus (Clavispora lusitaniae), Spom (Schizosaccharomyces pombe), Hsap (Homo sapiens), and Dmel (Drosophila melanogaster). (PDF) [file pgen.1003935.s001.pdf]

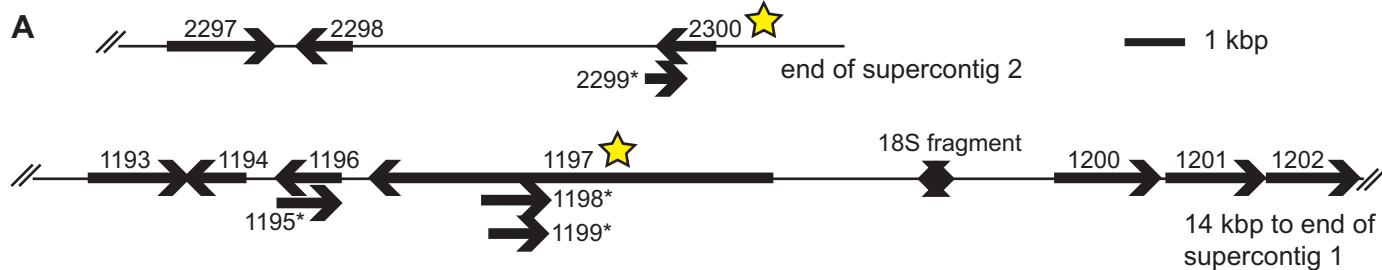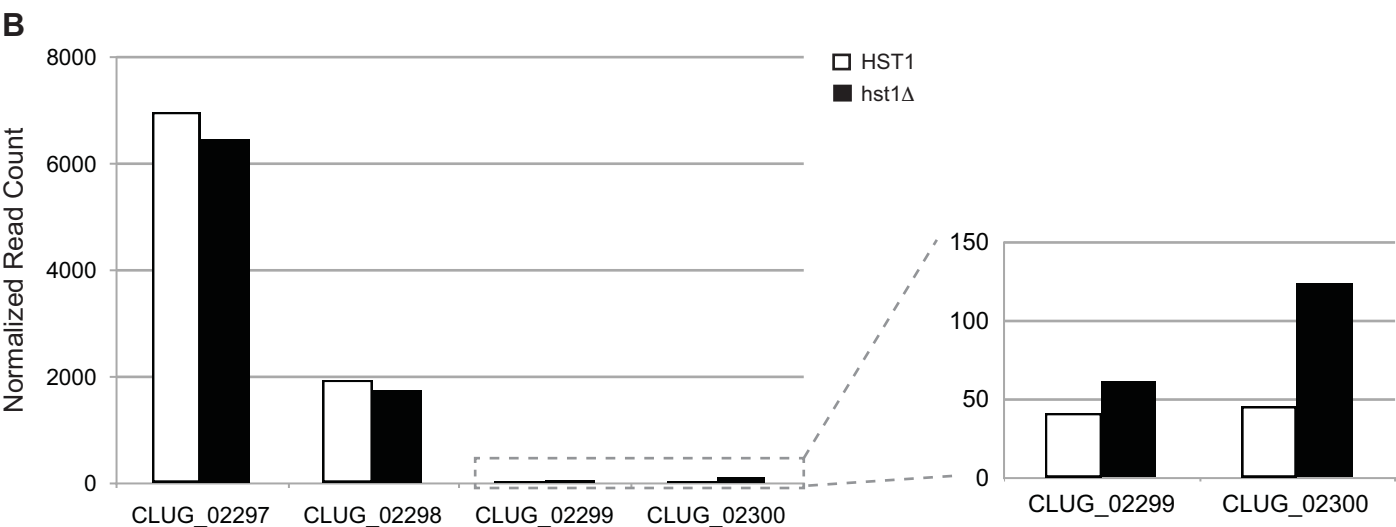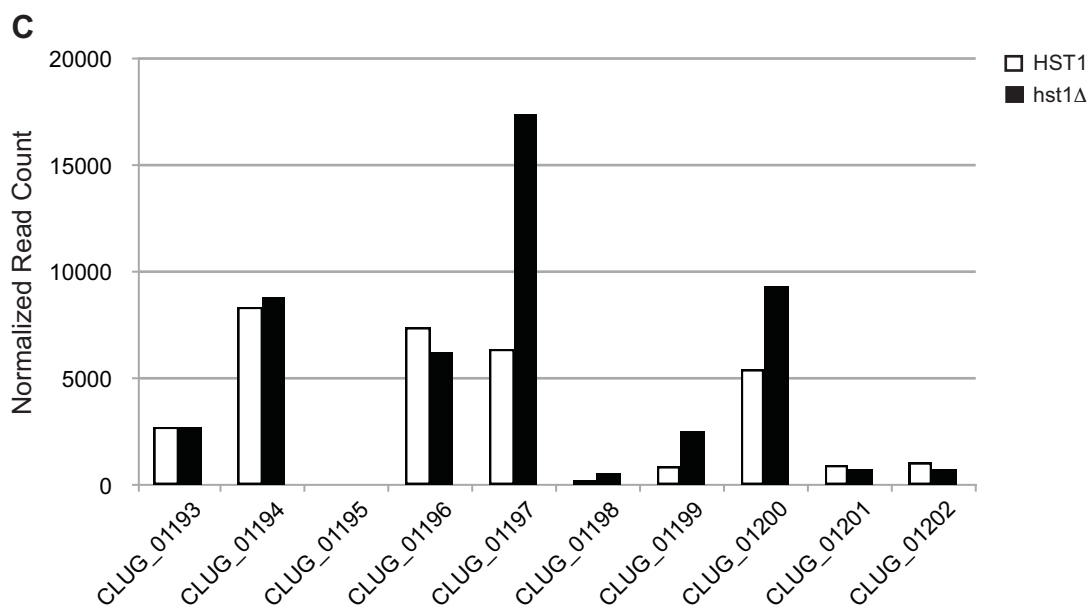

Supplement: Figure S2 — Induced genes near chromosome ends. (A) Diagrams display the genetic features surrounding two genes induced in hst1Δ cells compared to wild-type cells (indicated with stars). Genes marked with asterisks are dubious because they lack similarity to known proteins in Genbank and are antisense to bona fide genes. (B) Average normalized read counts derived from RNA-seq experiment are plotted for genes located near the right end of supercontig 2. (C) Normalized read counts are plotted for genes located near the right end of supercontig 1. (PDF) [file pgen.1003935.s002.pdf]

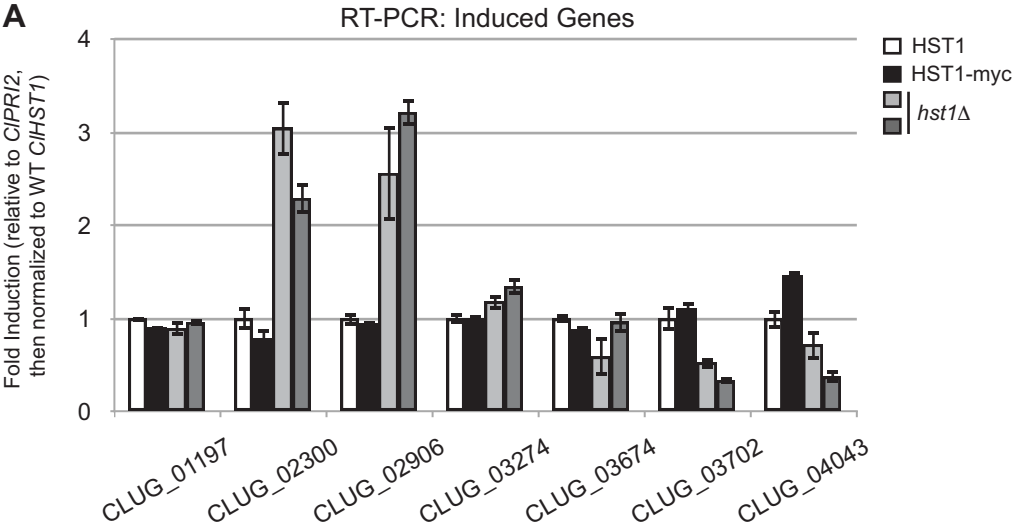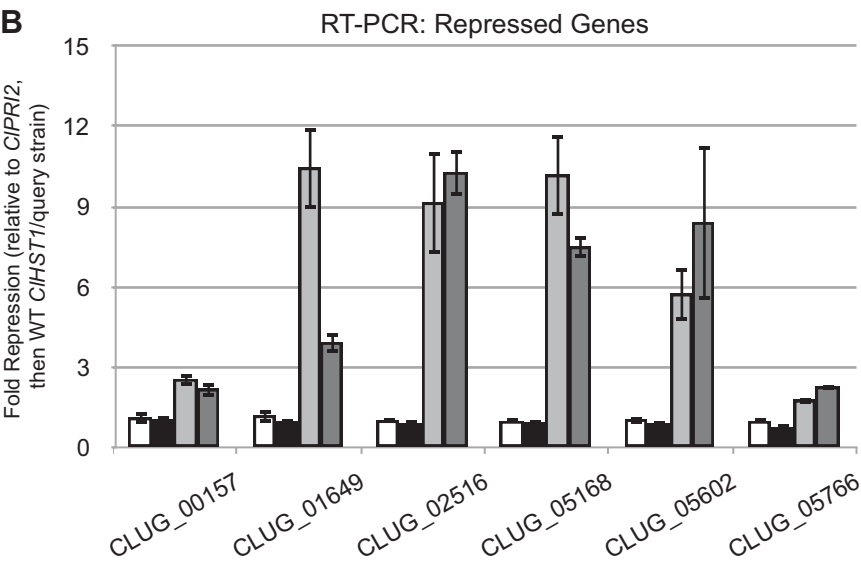

Supplement: Figure S3 — The expression of Hst1-influenced genes is the same in wild-type and HST1-myc strains. (A) Expression was assessed by quantitative RT-PCR in ClHST1 (LRY2826, white), ClHST1-myc (LRY2858, black) and two independently constructed Clhst1Δ strains (LRY2671; medium gray; LRY2672, dark gray). Levels of mRNA for each gene were first normalized to ClPRI2 (CLUG_00368) and then expressed relative to the WT ClHST1 strain. (B) Expression of genes repressed in hst1Δ strains was measured as in part A. For fold repression, the inverse of the fold expression was calculated. (PDF) [file pgen.1003935.s003.pdf]

# Sir2 and Hst1 proteins from CTG clade species

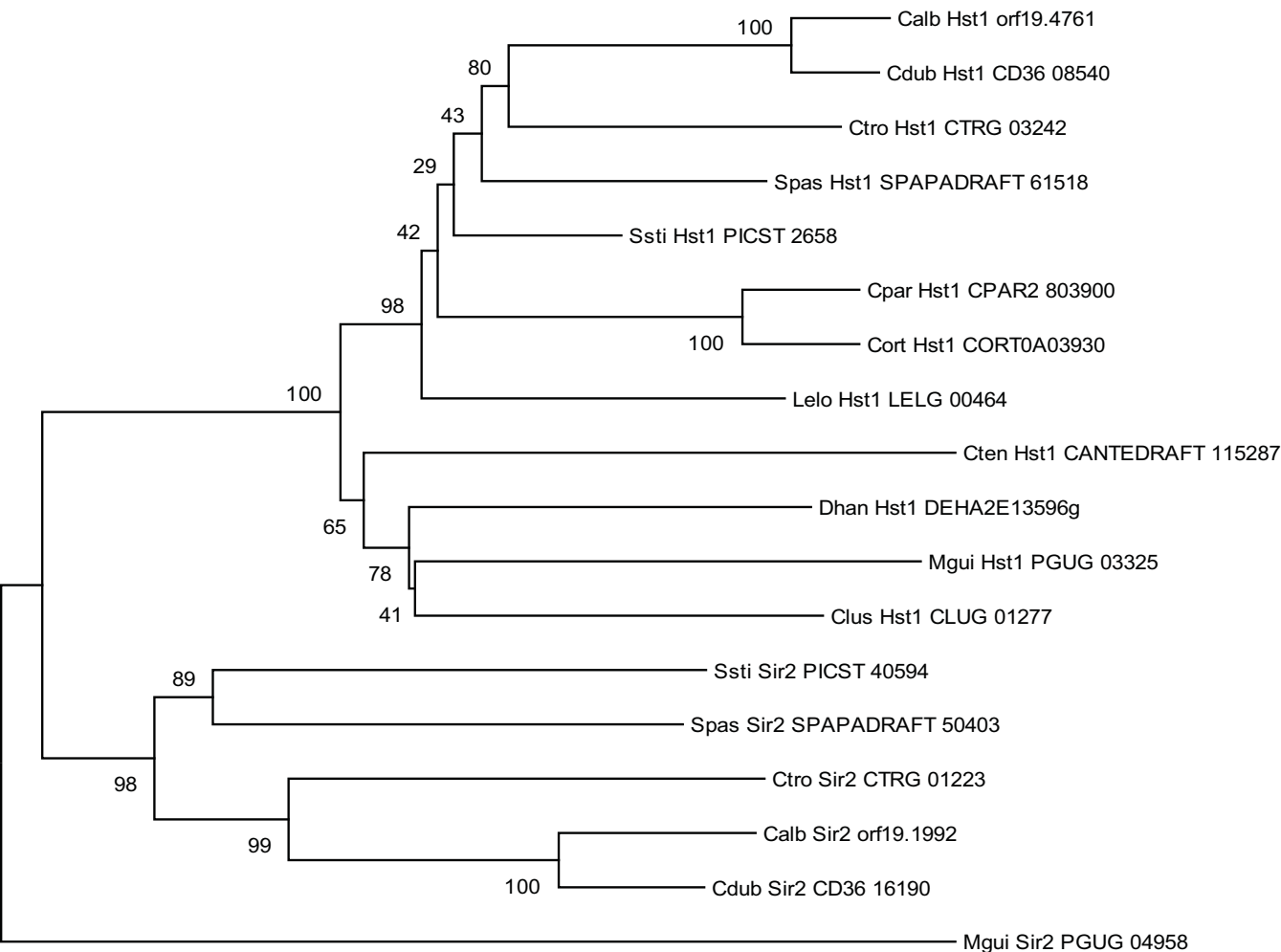

0.1

Supplement: Figure S4 — Phylogenetic tree of Sir2 and Hst1 proteins from CTG clade species. A phylogenetic tree represents the relationships of the core catalytic domains of Sir2 and Hst1 proteins from CTG clade species. The tree was constructed using the CLUSTAL_W algorithm of MEGA version 4.0 [67], and distances were calculated with default parameters of the neighbor-joining algorithm applying amino: poisson correction [68] in a pair-wise deletion procedure. The robustness of tree topologies was evaluated by 500 bootstrap replications. Sir2 and Hst1 from the following species were included: Calb (Candida albicans), Cdub (Candida dubliniensis), Ctro (Candida tropicalis), Spas (Spathaspora passalidarum), Ssti (Schefferomyces stipitis), Cpar (Candida parapsilosis), Cort (Candida orthopsilosis), Lelo (Lodderomyces elongisporus), Cten (Candida tenuis), Dhan (Debaryomyces hansenii), Mgui (Meyerozyma guilliermondii), and Clus (Clavispora lusitaniae). (PDF) [file pgen.1003935.s004.pdf]
