# Supplementary material for: The Deacetylase Sir2 from the Yeast Clavispora lusitaniae Lacks the Evolutionarily Conserved Capacity to Generate Subtelomeric Heterochromatin
Source: PLoS Genet. 2013 Oct 31;9(10):e1003935. doi: 10.1371/journal.pgen.1003935 (PMC3814328; doi:10.1371/journal.pgen.1003935)
Supplement: Table S1 — Oligonucleotides used for ChIP. The sequences of oligonucleotides used for chromatin IP experiments are provided. (PDF) [file pgen.1003935.s005.pdf]

Table S1. Oligonucleotides used for ChIP

C. lusitaniae Target	Sequence
ClPRI2 (CLUG_00368)	CGGAGCAAATCGCAAGTACC
	GTGTTGTTGAAGACGCGTAGTG
**rDNA**	
NTS1	CATACGACTTAGATGTACAACGGAG
	GTTGGGCGTCACTTGGC
NTS1	GATCTGGAGAATAGGAATTAAGTGG
	CTTGTATTTTGCATCTTTCGCCTG
NTS1	GCAAATACAATGAAGCGGTCATCAG
	CCTGGTGTTGTATCGCCTG
8S gene promoter	GACTAACGTTGATCGGACGG
	CCAATCCCTCCGCAACTATATAAACC
NTS2	CTCGGATCAAGCCCTAATTAGG
	GGCACACCACAAAATTACGAAAACG
NTS2	GCCTCATCGGGGGAAAAAAC
	GTTCGTCGCTAACATTTTTATGGACG
NTS2	GACAATGTCAGGGCGAGC
	CAGGGTGCGAGAATATACCG
18S gene	CTGCGAAAAGCATTTGCCAAGG
	CTTGCGACCATACTCCCC
Junction between 5.8S and 26S genes	CTCGAGGCATTCCTCGAG
	CTGGGGCAATCCCTGTTG
26S gene	GGAAACTCTGGTGGAGGC
	GGCTAAGGCTATTTCCGCC
26S gene	CTCCAAGGTTAACAGCCTCTAG
	GGGTTGGCAAGCAAGCAC
26S gene	GCTCTTCCTATCATACCGAAGC
	GGTGCCTGATCAGACAGG
**Telomeres**	
Telomere 2L	GCACCTGAGTTGGTTTCACAG
	GCTTTTCGTGACAATTGACATGGC
Telomere 4R	GGTTTCTCGATTGTTGACGACTC
	CAGTAAGCGAGTCTTCCAGTG
Telomere 5L	CGTGTGAGCAATGGGTTGTAAAC
	GTTAGCAAGGAATTGACTCCTAGTC
Telomere 7L	CCTAGTCGGGCACCTAAG
	CCGTCCCCTCACTAAAAACAAAC
Telomere 7R	GCCCTCTTCATACTCTACAACG
	CGCATTTGGAAAGAGGTTGATATC

**Gene Promoters**	
CLUG_01197	GCGAGTTTCGCGTAAGTAGC
	CGAACACTGGAACGGTAACG
CLUG_02300	CCAACACAGAAGCAATTGTCACC
	GGAAAACAGGGAGCGAAGC
CLUG_02516	GACTTTGTCCGAGACACCG
	CTCGTAGTTGGTGTTCTGCG
CLUG_02906	CCCCTCTAAAAGCATGTAGTACAC
	CTGAAGGAGAAGCCGGAG
CLUG_03274	GCGCTGATAACGGCTCAG
	GAACGGGCAGAAAACGGAAAC
CLUG_05602	CCGTACATCATCAATGTAGTGGC
	CAGTAAGGGCTGAACTCGC
