# Supplementary material for: The Deacetylase Sir2 from the Yeast Clavispora lusitaniae Lacks the Evolutionarily Conserved Capacity to Generate Subtelomeric Heterochromatin
Source: PLoS Genet. 2013 Oct 31;9(10):e1003935. doi: 10.1371/journal.pgen.1003935 (PMC3814328; doi:10.1371/journal.pgen.1003935)
Supplement: Table S2 — Oligonucleotides used for RT-PCR. The sequences of oligonucleotides used for reverse transcriptase- qPCR analyses are provided. (PDF) [file pgen.1003935.s006.pdf]

Table S2. Oligonucleotides used for RT-PCR

C. lusitaniae **Target**	**Sequence**
ClPRI2 (CLUG_00368)	GCCCACGGATGTCCTTAC
	CAACTGCCTCGACCTGTC
CLUG_01197	GTCTGGACAATCCACTGCG
	GACTGGCGACAGAAAGACG
CLUG_02300	GTCCTACACAGACCCATCG
	CTTGTGGCGGAACAACCG
CLUG_02906	GCTCCGGCATTGGCATTG
	CTTAGCGACCTCTTGTTCCC
CLUG_03274	GACTCAAACACCAACGGGTC
	GGAAACGCCAGTGACGAC
CLUG_03674	GCTATCCTCCGCACTGTC
	GCCCCATCTACGCAACATC
CLUG_03702	GGCATAGGTCAGGCCAATTC
	GGACACTTGAGGGTAGGG
CLUG_04043	CCACAAGGTAACTGGTCTCC
	GCGCCGTTCATCTCGTTG
CLUG_00157	CTTGGATGTGAAGTACCACAAGC
	GGTCGCTAGTTTCCTTTGGAAC
CLUG_01649	GGGTCAGTTGATGGGAATGG
	CATCATTTCAGCTCCAACGGC
CLUG_02516	CACACGTAATGTGCTCCGG
	GGACCTTACTACGTGGAGTTG
CLUG_05168	GTCAACTCCCTGCTGTTTGG
	CTCCAATGTCTGTCTGCGTC
CLUG_05602	GGTACGACTGATCCCAAAACC
	CAGTCCAACGACCCACAG
CLUG_05766	CCATTCCACTGGAAGGCTAC
	GCTCCTTCTTGATGTTTGCGG
