# Supplementary material for: The Deacetylase Sir2 from the Yeast Clavispora lusitaniae Lacks the Evolutionarily Conserved Capacity to Generate Subtelomeric Heterochromatin
Source: PLoS Genet. 2013 Oct 31;9(10):e1003935. doi: 10.1371/journal.pgen.1003935 (PMC3814328; doi:10.1371/journal.pgen.1003935)
Supplement: Table S3 — Oligonucleotides used to generate probes. The sequences of oligonucleotides used to generate probes for Southern analysis are provided. (PDF) [file pgen.1003935.s007.pdf]

**Table S3. Oligonucleotides used to generate probes**

| *C. lusitaniae* Target | Sequence |
|---|---|
| 2L probe | GCACCTGAGTTGGTTTCACAG |
| | GCAGGCATCTGGGAGTTG |
| 4R probe | GGGTGACTCAACTCGGGT |
| | GTCACCCACATACATTGGCC |
| 5L probe | CGTGTGAGCAATGGGTTGTAAAC |
| | GCAAAAAGTGGCGACTGACG |
| 7R probe | GCCCTCTTCATACTCTACAACG |
| | GTCTGTGAAGTCAGCCAATTGC |
| 7L probe | CCTAGTCGGGCACCTAAG |
| | CAAGTCCACTGTTCCGAATGG |
